# Photobiomodulation for the management of inferior alveolar nerve paresthesia after implant surgery: A randomized clinical trial

**DOI:** 10.4317/medoral.27602

**Published:** 2025-11-22

**Authors:** Mohammad Gerayeli, Javad Sarabadani, Morteza Jalaiean-Nasrabadi, Zeinab Ghasemi

**Affiliations:** 1 Department of Periodontics, School of Dentistry, Mashhad University of Medical Sciences, Mashhad, Iran; 2 Oral and Maxillofacial Medicine Department, Oral and Maxillofacial Diseases Research Center, School of Dentistry, Mashhad University of Medical Sciences, Mashhad, Iran; 3 Dentist, Mashhad, Iran

## Abstract

**Background:**

Photobiomodulation (PBM) has shown promise for managing nerve paraesthesia. This trial assessed the efficacy of an 810-nm diode laser on deep-mechanical, superficial-mechanical and thermal sensitivity for inferior alveolar nerve paresthesia after implant surgery.

**Material and Methods:**

Twenty-four adults with recent implant-related paraesthesia were randomly assigned, in a parallel design, to an intervention or control group; both groups received routine vitamin-B supplementation. The intervention group underwent eight diode-laser sessions (200 mW power and 6 J/cm2 energy density) over four weeks, directed at peri-implant mucosa and adjacent cheek skin. The control group attended identical sessions with an inactive laser. Blinded examiners recorded visual-analogue-scale (VAS) scores for the Clamp (deep mechanical), Swab (light mechanical) and Ice (thermal) tests at baseline and at two and four weeks after the final session. Data were analysed with the Shapiro-Wilk test, independent-samples t-test and Friedman test (=0.05).

**Results:**

All twenty-four randomised participants (mean age 51±7 years) completed follow-up. Baseline VAS scores did not differ between groups (p&gt;0.44). Although both groups improved over time, the VAS scores for paresthesia reductions in the PBM group were significantly greater than those in the control group at both follow-ups for all three tests (all p&lt;0.001). No adverse events were reported.

**Conclusions:**

Eight sessions of 810-nm PBM produced faster and more pronounced sensory recovery than sham treatment in patients with implant-related paraesthesia.

## Introduction

Paresthesia of the inferior alveolar nerve (IAN) represents a well-documented complication arising from mechanical or thermal injury, traction, or compression of the nerve during common dental procedures. It has been reported following mandibular third molar extraction, endodontic therapy, orthognathic surgery, local anesthetic injection, and dental implant placement. After mandibular third molar surgery, transient and permanent IAN injuries have been observed in approximately 1.20% and 0.28% of cases, respectively (1). For dental implants, the reported short-term and long-term incidences are about 11% and 3%, respectively (2). Resultant sensory disturbances range from mild hypoesthesia to complete anesthesia and are frequently accompanied by paresthetic sensations such as tingling, burning, or numbness. These neurosensory deficits can markedly affect essential oral functions-including mastication, speech, and swallowing-and may substantially diminish patients' overall quality of life. In severe or persistent cases, the associated functional impairment and discomfort can also lead to psychological distress and, occasionally, medicolegal claims (1 , 2).

Conventional management of peripheral nerve injury remains largely based on clinical experience. Mild forms such as neuropraxia or axonotmesis are typically managed conservatively using neurotropic agents-most commonly vitamin B12 and uridine combinations-together with physiotherapy or expectant observation. Combined supplementation with uridine nucleotides and vitamin B12 has been shown to support peripheral nerve repair by accelerating axonal conduction, enhancing myelin synthesis, and stimulating cellular processes essential for neural regeneration (3). In contrast, complete neurotmesis usually requires microsurgical intervention (4). Even with meticulous microsurgical repair, however, complete sensory recovery is unpredictable (4), and pharmacologic therapy often shortens symptom duration without restoring normal function.

This therapeutic uncertainty has prompted increasing interest in laser-based approaches that may enhance healing through photothermal or photochemical mechanisms (5). Among these, photobiomodulation (PBM) is defined as a form of light therapy that uses non-ionizing light sources-such as lasers or light-emitting diodes (LEDs)-to deliver low-energy red or near-infrared light in a non-thermal manner, thereby activating physiological mechanisms and cellular responses (6). The absorbed light enhances mitochondrial electron transport and ATP synthesis, triggering downstream pathways that promote axonal regeneration, remyelination, angiogenesis, and anti-inflammatory signaling while simultaneously reducing pain (6). Experimental and early clinical studies have demonstrated accelerated nerve conduction, reduced fibrotic scar formation, and upregulation of neurotrophic factors (7 - 9). Importantly, when applied within established therapeutic parameters, PBM has shown an excellent safety profile, with no adverse effects reported in head and neck tissues (6), although standardized treatment protocols are still lacking.

Therefore, the objective of this randomized, double-blind clinical trial was to evaluate whether adjunctive low-level laser therapy, combined with standard vitamin B and neurobion uptake, enhances sensory recovery of the IAN. Specifically, the study aimed to determine if PBM leads to superior improvements in deep mechanical (Clamp), superficial mechanical (Swab), and thermal (Ice) sensitivity scores compared with a passive laser control.

## Material and Methods

The protocol for this triple-blinded randomized controlled trial (RCT) was approved by the Ethics Committee of Mashhad University of Medical Sciences (approval code: IR.MUMS.DENTISTRY.REC.1403.076). Additionally, the protocol was registered with the Iranian Registry of Clinical Trials (IRCT) under the registration code: IRCT20231213060351N1, and it adheres to the CONSORT 2010 guidelines. The study was conducted from October 2024 to January 2025.

Sample size calculation

According to the findings of a previous study (3), which compared the effects of PBM and mecobalamin on the improvement of subjective symptoms of IAN injury-assessed using the visual analogue scale (VAS) following the extraction of impacted mandibular third molars-and based on a power of 80% and a significance level of =0.05, while accounting for an anticipated 30% dropout rate, the required sample size was calculated to be 12 patients per group (a total of 24 participants).

Participants

**Figure 1:**
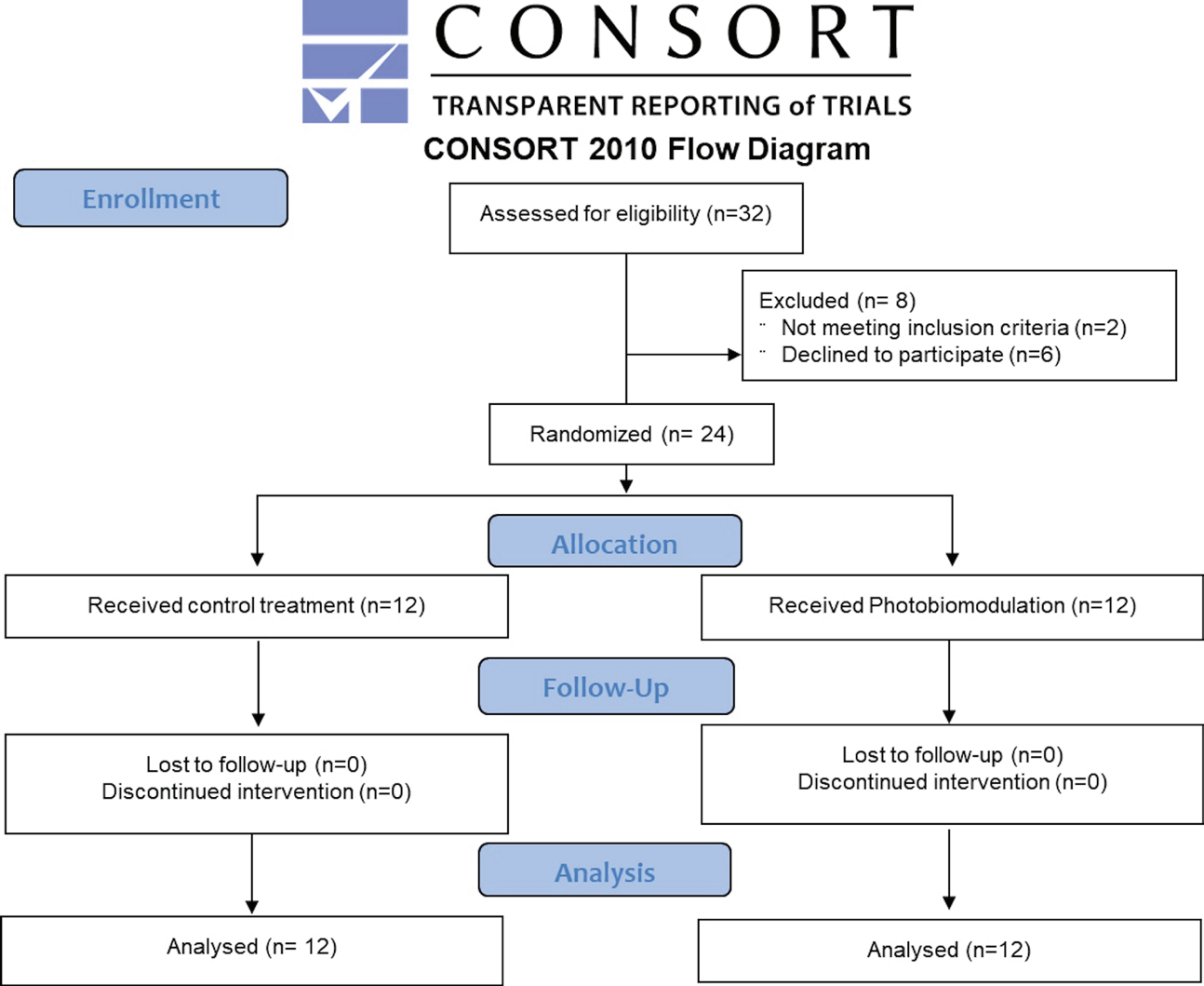
The CONSORT diagram of the study.

Adult patients who developed unilateral IAN paresthesia within three months after implant surgery and sought treatment at Mashhad Dental School were included in the study. The implant was removed prior to the treatment. Patients were excluded if they had any pre-existing neurosensory deficits or paresthesia prior to the implantation, had received medical, surgical, or laser treatments for the current injury, or had any systemic or neurological disorders. Other exclusion criteria included the use of antibiotics, anti-inflammatory medications, photosensitizing drugs, or neuroactive substances, as well as smoking, alcohol or substance abuse, and pregnancy or lactation. The objectives of the study was explained to the patients and written informed consent forms were received. Figure 1 shows the CONSORT diagram of the study.

1

Randomization and blinding

A variable-block randomisation list was produced with Random Allocation Software 2.0 by an independent researcher. Allocation codes were concealed in sequentially numbered, opaque, sealed envelopes. Each arm balanced for sex and side of paresthesia.

Participants, outcome assessors and data analyser were blinded to assignment. The laser operator (Z.G.) found out about the allocation only after opening the envelope immediately before the first session.

Interventions

Both groups were provided with routine supportive therapy consisting of oral mecobalamin (0.5 mg, three times daily; Eisai China Inc., Shanghai, China) (3) and Neurobion® injections (twice weekly for 3 weeks; Merck KGaA, Darmstadt, Germany).

Active PBM: Patients were treated using an 810 nm diode laser (Thor DD2; Thor Photomedicine Ltd, London, UK) with the parameters summarized in Table 1. The laser was applied in contact mode with slow, overlapping scanning across the implant placement site and the regions affected by paresthesia.

Table 1

Sham-laser (control group): The same hand-piece was applied for identical durations, but the emission was disabled; an internal light-emitting diode provided visual feedback so that the device appearance and operator actions matched the active procedure.

Each participant underwent eight sessions (two sessions per week with a 3-day interval for four consecutive weeks).

Outcome measures

Outcome evaluations were conducted at baseline (prior to the first treatment session), two weeks after the final session, and one month post-treatment by a calibrated examiner (M.J.N.) blinded to group allocation, under the supervision of a specialist in Oral and Maxillofacial Diseases (J.S.). Patients marked paresthesia intensity on a 10-cm line anchored at 0 ("no sensation disturbance") and 10 ("complete numbness") at each timepoint.

Clamp test (deep mechanical sensitivity): A light-pressure artery clamp was gently closed on the lower-lip mucosa at three standardized points. At each site, patients rated the perceived force on a VAS. This procedure parallels established orofacial neurosensory testing paradigms that utilize graded mechanical stimuli and subjective intensity ratings to detect changes in mechanosensitivity (10).

Swab test (superficial mechanical sensitivity): Light tactile sensitivity was assessed by stroking each site twice with a sterile cotton swab. Participants reported touch perception and rated the intensity using a VAS, consistent with established orofacial sensory testing methods (11).

Ice test (thermal sensitivity): Cold sensitivity was evaluated by applying a cotton-tipped applicator cooled in ice water (0 °C) to each test site for 2 seconds, after which subjects rated the perceived cold intensity on a VAS (12).

Statistical analysis

Data were analysed with SPSS v23 (IBM, Chicago, IL, USA). Data analysis was performed using independent samples t-test. Statistical significance was set at p&lt;0.05.

## Results

Each group comprised six men and six women. The mean age of participants in the control group was 51±5.67 years, which was statistically comparable to that of the intervention group (51.25±7.78 years; p=0.872).

Table 2 presents the comparison of VAS scores between the control and intervention groups at each assessment point for the clamp, swab, and ice tests. At baseline, there were no statistically significant differences in mean VAS scores between the groups across all tests. Two weeks after treatment, the intervention group showed a marked reduction in pain intensity compared with the control group for all three tests: Clamp (p&lt;0.001), swab (p&lt;0.001), and ice (p=0.004). This trend persisted at four weeks post-treatment, where the intervention group continued to demonstrate significantly lower VAS values than the control group for the clamp (p&lt;0.001), swab (p&lt;0.001), and ice (p=0.002) tests.

Table 2

Table 3 summarizes the changes in VAS scores between time intervals. Between baseline and two weeks, the intervention group demonstrated significantly greater reductions in VAS scores compared with the control group for all tests:

Table 3

Clamp (3.00±1.21 vs. 0.58±0.51; p&lt;0.001), swab (3.58±1.97 vs. 0.58±0.90; p&lt;0.001) and ice (1.91±0.66 vs. 0.25±0.75; p&lt;0.001).

From two to four weeks, no significant differences were observed between groups (p&gt;0.05). Considering the entire study duration (baseline to four weeks), the intervention group showed significantly greater reductions in VAS scores for the clamp (3.36±1.23 vs. 1.25±0.62; p&lt;0.001), swab (4.50±1.83 vs. 1.33±1.23; p&lt;0.001), and ice (2.25±0.75 vs. 0.66±0.98; p&lt;0.001) tests compared with the control group.

## Discussion

The present study evaluated the efficacy of PBM therapy combined with supportive care in the management of IAN paresthesia following implant placement.

The results demonstrated that PBM at 810 nm significantly enhanced deep-mechanical, superficial-mechanical, and thermal sensitivity compared with supportive care alone. These findings are consistent with previous clinical reports supporting the use of red-to-near-infrared laser therapy as an effective adjunct in the treatment of IAN paresthesia (13 , 14). Khullar et al. (13) reported successful recovery using 820 nm irradiation even in cases where the nerve injury had persisted for more than six months. Similarly, Miloro et al. (14) observed significant improvement within 14 days when PBM was initiated as early as six hours after surgery. A systematic review of laser-acupuncture for dento-alveolar neuropathies by Manente et al. also found that red-to-near-infra-red wavelengths between 790 and 810 nm, delivered at least twice weekly, yielded consistent gains in facial sensory and motor recovery, although methodological heterogeneity existed (15). According to the systematic review by Nasiri et al. (16) PBM at 808-810 nm applied for five to ten sessions markedly improved the rate of neurosensory recovery. Nonetheless, substantial heterogeneity persists across studies, primarily because of variations in study design and the absence of standardized treatment parameters.

The greatest improvement in the intervention group occurred between baseline and the two-week evaluation. This suggests that PBM primarily accelerates the early reparative phase of neural recovery-a finding consistent with other clinical trials that concentrated treatment sessions within the first month and reported similarly rapid functional gains (17 , 18).

Improvement in the control group likely reflects the natural course of IAN recovery, assisted by adjunctive vitamin supplementation. Spontaneous sensory restitution is well documented, particularly in milder nerve injuries (neurapraxia or mild axonotmesis). For example, in a large retrospective study of lower third molar extractions, temporary inferior alveolar neurosensory changes occurred in about 1.75% of patients and permanent changes in 0.71%, with cumulative recovery rates of 25.0%, 60.1%, and 71.1% at one, three, and six months, respectively (19). Other reports of implant- and nerve-injury cohorts have observed transient neurosensory disturbance rates between 15-40% and permanent deficit rates up to 5% (20). Thus, the gradual improvement seen in the control arm aligns with expected spontaneous healing supported by adjunctive vitamin supplementation, while the PBM arm exhibited a faster and more pronounced recovery.

Evidence suggests that extending the duration of PBM therapy can compensate for a delayed initiation. For example, Mobadder et al. (21) reported complete recovery of a six-month-old nerve injury after 42 laser sessions. In contrast, another study that commenced PBM immediately following complex odontoma removal observed substantial VAS improvement by the tenth session but minimal additional benefit beyond the seventh (22). Therefore, eight-sessions of PBM therapy was considered in the present study to maximise therapeutic effect.

The primary mechanism underlying the therapeutic effects of PBM involves absorption of near-infrared photons by cytochrome-c oxidase (Complex IV) within the mitochondrial respiratory chain. This interaction enhances electron transport, mitochondrial membrane potential, and oxidative phosphorylation, thereby increasing ATP production-a critical energy source for axonal repair, remyelination, and neuronal survival (6). The 810 nm wavelength also lies within the tissue optical window, allowing sufficient penetration to reach deeper neural structures such as the IAN (6 , 23).

Beyond mitochondrial stimulation, PBM influences several cellular signaling pathways. Lasers in this wavelength range can modulate ion channel activity and neurotransmitter release, thereby contributing to the neuromodulatory regulation of nerve signaling and synaptic function (7). Moreover, PBM modulates the cellular redox state and nitric oxide signaling, enhancing microcirculation, angiogenesis, and cellular homeostasis (8). It also exerts potent anti-inflammatory and immunomodulatory effects by downregulating pro-inflammatory cytokines (such as TNF-, IL-1, and IL-6) and promoting macrophage polarization from the M1 (pro-inflammatory) toward the M2 (reparative) phenotype, thereby fostering an environment conducive to nerve regeneration. Additionally, PBM has been shown to upregulate neurotrophic factors (e.g., BDNF, NGF) and growth-associated proteins (e.g., GAP-43), further supporting axonal outgrowth and remyelination (8 , 9). Given that the present study employed a low power density (&lt; 50 mW/cm²), thermal effects were negligible, reinforcing the concept that PBM exerts its therapeutic effects predominantly through biostimulatory photochemical mechanisms rather than photothermal heating (24).

While several previous studies have reported no significant association between demographic variables-such as gender-and the recovery of paresthesia following laser therapy (25 , 26). Other investigations have identified a positive correlation between younger age and higher rates of sensory improvement (27 , 28), likely because aging impairs key regenerative mechanisms. In older individuals, Schwann cells become less efficient at reprogramming into repair mode, delaying myelin clearance and reducing support for axonal regrowth (29). Moreover, aging is characterized by a more pro-inflammatory microenvironment, delayed macrophage activation, and diminished angiogenic capacity (30). Accordingly, in the present study, the intervention and control groups were matched for demographic characteristics to minimize potential confounding effects.

The present study had some limitations. It was conducted at a single center with a follow-up period limited to one month. Participants were not stratified by the severity of nerve injury, and objective electrophysiological assessments were not performed. Future investigations involving larger, multicenter cohorts, longer follow-up durations, and direct comparisons of different energy densities, wavelengths, and conservative treatment modalities are recommended to determine the optimal parameters and long-term effectiveness of PBM therapy.

## Conclusions

Within the limitations of this study, eight sessions of 810 nm PBM therapy appear to accelerate sensory recovery following implant-induced inferior alveolar nerve paresthesia, supporting its use as an adjunct to standard postoperative care.

## Figures and Tables

**Table 1:** Table PBM parameters.

Parameter	Specification
Laser type	Diode laser (Thor DD2; Thor Photomedicine Ltd, London, UK)
Wavelength	810 nm
Mode of operation	Continuous
Average output power	200 mW
Spot size	1 cm²
Power density	0.2 W/cm²
Energy density (fluence)	6 J/cm²
Exposure duration	30 s per point
Sessions	8 sessions (twice weekly for 4 weeks)
Accumulated energy per session	approximately 48 J

1

**Table 2:** Table Comparison of VAS between groups, in each timepoint.

Measurement	Group	Pre-treatment		Two weeks post-treatment		Four weeks post-treatment	
		Mean ± SD	P-value	Mean ± SD	P-value	Mean ± SD	P-value
Clamp test	Control	3.92 ± 1.08	0.729	3.33 ± 1.073	< 0.001*	2.67 ± 0.98	< 0.001*
	Intervention	4.08 ± 1.24		1.08 ± 1.165		0.42 ± 0.52	
Swab test	Control	5.00 ± 1.31	0.813	4.33 ± 0.88	< 0.001*	3.58 ± 0.90	< 0.001*
	Intervention	5.08 ± 2.02		1.50 ± 1.44		0.58 ± 0.66	
Ice test	Control	2.00 ± 0.83	0.444	2.00 ± 0.99	0.004*	1.50 ± 1.00	0.002*
	Intervention	2.50 ± 1.00		0.58 ± 0.79		0.25 ± 0.45	

SD: Standard deviation. *Values less than 0.05 indicate a significant difference among the groups using independent sample t-test.

**Table 3:** Table Comparison of VAS changes over study intervals between groups.

Measurement	Baseline to 2 weeks				2 to 4 weeks		Baseline to 4 weeks		
	Control	Intervention	P value	Control	Intervention	P value	Control	Intervention	P-value
	Mean ± SD	Mean ± SD		Mean ± SD	Mean ± SD		Mean ± SD	Mean ± SD	
Clamp test	0.58 ± 0.51	3.00 ± 1.21	< 0.001*	0.66 ± 0.49	0.50 ± 0.77	0.755	1.25 ± 0.62	3.36 ± 1.23	< 0.001*
Swab test	0.58 ± 0.90	3.58 ± 1.97	< 0.001*	0.75 ± 0.62	0.91 ± 1.08	0.977	1.33 ± 1.23	4.50 ± 1.83	< 0.001*
Ice test	0.25 ± 0.75	1.91 ± 0.66	< 0.001*	0.41 ± 0.51	0.33 ± 0.49	0.843	0.66 ± 0.98	2.25 ± 0.75	< 0.001*

SD: Standard deviation. *Values less than 0.05 indicate a significant difference among the groups using independent sample t-test.

## Data Availability

Data underlying the results of this study are available from the corresponding author on reasonable request.
